# Effect of Various Carbohydrates in Aqueous Solutions on Color Stability and Degradation Kinetics of Selected Anthocyanins During Storage

**DOI:** 10.3390/foods13223628

**Published:** 2024-11-14

**Authors:** Adam Tobolka, Tereza Škorpilová, Filip Beňo, Tereza Podskalská, Aleš Rajchl

**Affiliations:** Department of Food Preservation, Faculty of Food and Biochemical Technology, University of Chemistry and Technology in Prague, Technická 5, Dejvice, 166 28 Prague, Czech Republic; adam.tobolka@vscht.cz (A.T.); tereza.skorpilova@vscht.cz (T.Š.); filip.beno@vscht.cz (F.B.); tereza.podskalska@vscht.cz (T.P.)

**Keywords:** anthocyanins, thermal stability, color change, sugars, degradation kinetics, accelerated storage test

## Abstract

Anthocyanins are flavonoid substances of plant origin with potential antioxidant effects. Because of their intense colors, they are used as natural dyes in food. However, their stability in food matrices is limited. This study aimed to verify the effect of selected carbohydrates on the stability of anthocyanins (cyanidin-3-*O*-*β*-glucopyranoside, cyanidin-3-*O*-*β*-galactopyranoside, cyanidin-3-*O*-*β*-rutinoside and delphinidin-3-*O*-*β*-rutinoside) during the accelerated storage test, since carbohydrates help to preserve the typical color of anthocyanins, increase their shelf-life and availability in the organism, and reduce losses during processing. Moreover, the kinetic parameters of anthocyanin degradation (E_a_, *k*, t_1/2_) were determined. Sucrose was found to have the greatest potential for retarding anthocyanin degradation during storage, whereas fructose exerted an accelerating effect. Glycosidation of anthocyanin aglycone had no significant effect in terms of their stability. Anthocyanin degradation was significantly positively correlated with the change in the a* parameter (redness), and subsequently, a significant positive correlation was observed in the determination of the kinetic parameters for anthocyanins and the a* parameter. The highest stability of anthocyanins was observed in the presence of sucrose and their degradation can be predicted by the value of the a* parameter, which would also be a very fast and non-destructive method for food processing companies.

## 1. Introduction

Anthocyanins are the most abundant group of hydrophilic plant dyes and form most of the flavonoids found in nature. They are found in fruits and vegetables, most commonly in berries, and are responsible for their characteristic colors [1]. Anthocyanins are glycosides whose aglycones form anthocyanidins. There are 17 different anthocyanidins found in nature, the most important of which are delphinidin, pelargonidin, peonidin, petunidin, malvidin and cyanidin [2]. As several different monosaccharides can be attached to individual anthocyanidins and can be substituted, the number of anthocyanins is multiplied by the number of anthocyanidins [3]. In the food industry, anthocyanins have been utilized for more than 100 years for coloring acidic foods [4]. However, they have also been investigated for their effects on human health, including antioxidant and anti-inflammatory properties, as well as effects on obesity, diabetes, tumors and ulcers [5]. Owing to the use of anthocyanins as an alternative to synthetic food dyes and their health benefits, chemical stability is a topic of interest. The stability of anthocyanins is relatively low and is influenced by factors such as temperature, pH, the presence of enzymes, exposure to oxygen, exposure to radiation, the structure of the molecule and the presence of other certain compounds, such as metal, ascorbic acid or sulfur dioxide [6]. Due to co-pigmentation, anthocyanins are stable in flowers of some plants, fruits and vegetables [7]. The limiting parameter for the use of anthocyanins in industries, such as food, pharmaceuticals and cosmetics, is their thermostability and also susceptibility to oxidation. Anthocyanins are relatively resistant to heating at a low pH. However, they are very unstable at high pH values [5]. A positive effect on the resistance of anthocyanins to degradation during heating is the addition of sugars, as well as the addition of metal ions such as ferric cation, intermolecular co-pigmentation with phenolic compounds (e.g., gallic acid, ferulic acid or other flavonoids), encapsulation by biopolymers of proteins or polysaccharides, addition of yeast mannoproteins, fortification with thiol-based antioxidants such as cysteine, addition of Maillard reaction products and mild blanching to inactivate enzymes and exclude oxygen during heating [5].

The addition of sugars can reduce the activity of water and thus achieve a certain protective effect, which may be due to the inhibition of enzymatic reactions or the hindering effect on condensation reactions [8]. Meanwhile, a study by Jiménez et al. [9] reported the negative effect of lowering water activity with respect to anthocyanin content, where the reduction of water activity to 0.6 resulted in up to four times faster degradation of anthocyanins in blackberry juice samples. There is no clear opinion from the academic community regarding the effect of carbohydrates on anthocyanin stability. Some carbohydrates affect stability positively [8] and others negatively [10,11]. Sugars and high storage temperatures are the main factors in anthocyanin degradation in fruit and vegetable products [12]. Carbohydrate concentration is an important factor as there is extensive debate in the literature regarding which carbohydrate concentrations positively influences the slowing down of anthocyanin degradation in different matrices and vice versa. A study by Li et al. [13] investigating the effect of the sugars sucrose, glucose and fructose on the stability of purple yam anthocyanins reported that a carbohydrate concentration of 50 g L^−1^ increased anthocyanin degradation when heated to 80–100 °C at pH = 4. Nikkhah et al. [14] studied the effect of sugar content on the stability of anthocyanins. Sucrose exerted the greatest effect on the stability of anthocyanins at a concentration of 20%, with this protective effect diminishing at higher sucrose concentrations. Sugar concentrations higher than 20% had a stabilizing effect on the color of anthocyanins, mainly due to a reduction in water activity. Fruit nectars are usually sold at room temperature (20 °C), which can increase the rate of anthocyanin degradation. Thus, the type of carbohydrate added to nectars should be carefully chosen [15]. Previous studies have demonstrated that sugars (sucrose, fructose, glucose and honey) had a significant effect on anthocyanin stabilization in strawberry spreads [8], blood orange juice [11] or anthocyanin model systems [12].

As regards the determination of the color of fruit, one of the most widely employed methods of color determination by reflection of the surface of the sample is currently CIEL*a*b* [16]. In the food industry and scientific sectors, the CIEL*a*b* color measurement is recognized and used as an international standard system [17]. The system is given by three variables that define a three-dimensional space (sphere). Lightness occupies the vertical axis in the system and is represented by L* (100 = white; 0 = black). The other variables are a* (redness > 0; greenness < 0) and b* (yellowness > 0; blueness < 0) expressed by horizontal axes perpendicular to the L* axis [18,19,20]. Another possibility of the detected coordinates is to calculate the total color change (∆E_Lab_*), which enables us to determine the degree of color change with only one variable [21].

This study aimed to determine, using model anthocyanin samples in the environment of selected carbohydrates in aqueous solutions, whether any of the selected carbohydrates exhibit a significant protective effect on the selected anthocyanin and whether this effect is reflected in the overall color change (∆E_Lab_*) and retardation of red color degradation (a*) of the anthocyanin standards when stored at elevated temperatures during the accelerated storage test. Moreover, the kinetics of the degradation reactions of anthocyanins were determined for this study by calculating the parameters E_a_, *k* and *t*_1/2_ for each sample.

## 2. Materials and Methods

### 2.1. Preparation of Anthocyanin Standards

Four standards of anthocyanins were selected, namely, cyanidin-3-*O*-*β*-glucopyranoside (Cya3Glc) (>97%) (Polyphenols AS, Sandnes, Norway), cyanidin-3-*O*-*β*-galactopyranoside (Cya3Gal) (≥95%) (Merck, Darmstadt, Czech Republic), cyanidin-3-*O*-*β*-rutinoside (Cya3Rut) (>97%) (Polyphenols AS, Sandnes, Norway), and delphinidin-3-*O*-*β*-rutinoside (Del3Rut) (>97%) (Polyphenols AS, Sandnes, Norway). A mass concentration of 600 mg L^−1^ was chosen for the preparation of the anthocyanin standard stock solution. Weighing of the standard was performed using a Model MYA 5.5Y microbalance (Radwag, Radom, Poland) and a calibrated weighing crucible. After transferring the weighed anthocyanin into the volumetric flask, it was made up to the mark with methanol and water (1:3) according to the IFU (International Fruit and Vegetable Juice Association, Paris, France) No. 71 (1998) method. The stock solutions of anthocyanin standards were stored in a freezer at −18 °C, and the flasks were covered with aluminum foil before being used for the preparation of model samples.

### 2.2. Stock Solutions of Sugars

Stock solutions (25% *w*/*w*) of sucrose (Suc) (>99.5%) (Sigma-Aldrich, Darmstadt, Germany), glucose (Glc) (>99.5%) (Sigma-Aldrich, Darmstadt, Germany), fructose (Fru) (>99.5%) (Sigma-Aldrich, Darmstadt, Germany) and glucose/fructose (Fru/Glc) mixtures of 1:1 weight ratio were made by precipitating 125 g of sugar dissolved in 500 mL of distilled water adjusted to pH = 3.4 with acetate buffer (Millipore, Merck, Darmstadt, Germany). The concentration of sugar in the solution (°Brix) was determined using a digital refractometer (RM-40 Mettler Toledo, Prague, Czech Republic), and the water activity of the stock solutions was determined using an Aqualab 4TEV instrument (METER Group, Inc., Washington, DC, USA). The water activity was determined in each stock solution four times at 25 °C. To keep the pH constant at 3.4 in the samples, an acetate buffer was prepared from a 0.1 mol L^−1^ solution of acetic acid (99%) (Lach-Ner; Neratovice, Czech Republic) and a 0.1 mol L^−1^ solution of sodium acetate (99.5%) (Penta, Prague, Czech Republic) in the appropriate ratio (19:1).

### 2.3. Preparation of Samples for the Storage Test

The sugar solutions fortified with the anthocyanin standard for the accelerated storage test were made in a laminar box (SafeFAST Elite 212 D, FASTER Srl, Ferrara, FE, Italy) with vertical air flow with the sugar stock solution or the same addition of distilled water for the reference samples. The concentration of sugar solutions was selected based on previous experiments and the available literature where experiments were conducted with different concentrations of selected carbohydrates, and a concentration above 20% was selected as the appropriate concentration that caused slower degradation of some anthocyanins [4,22]. Using a refractometer, the Brix values were determined in the individual sugar solutions used for sample preparation. The resulting values were 25.02 ± 0.02 °Brix for Glc, 24.98 ± 0.03 °Brix for Fru, 25.01 ± 0.01 °Brix for Suc and 25.03 ± 0.04 °Brix for the Fru/Glc mixture. pH was measured using a SevenCompact pH meter (Mettler Toledo, Prague, Czech Republic) at 25 °C. The samples were prepared in dark glass vials due to the photolability of anthocyanins. To maintain the integrity of the experiment due to the different molar masses of the analytical standards of anthocyanins, the anthocyanins were converted to a substance concentration corresponding to 0.15 mol L^−1^ of the anthocyanin used in each vial. About 125 µL of the anthocyanin standard (Cya3Glc, Cya3Gal) at a concentration of 600 mg L^−1^ was pipetted into vials, and the remainder was made up with the appropriate sugar solutions and water at the same pH. The remaining two anthocyanins (Cya3Rut, Del3Rut) had different molar masses; therefore, a different volume of standards with the same initial stock solution concentration (600 mg L^−1^), namely, 166 µL, was pipetted, and the residue was made up of sugar solutions and water at the same pH. The total sample volume was adjusted to ensure that the dilution of each sample was uniform and the initial molar concentration was identical. Darkened glass vials covered with aluminum foil containing various sugar solutions fortified with the anthocyanin standards cyanidin-3-*O*-*β*-glucopyranoside, cyanidin-3-*O*-*β*-galactopyranoside, cyanidin-3-*O*-*β*-rutinoside and delphinidin-3-*O*-*β*-rutinoside were placed in thermostats at 20, 35, and 50 °C. The color change of the anthocyanins was determined once a week for 20 °C and 35 °C and twice a week for 50 °C. The total duration of the storage test was 4 weeks.

### 2.4. Determination of the Color Change

The reflectance of the stored sample was measured in cuvettes on a Minolta CM-5 benchtop spectrophotometer (Konica Minolta, Tokyo, Japan). The measured color of the sample was then recorded in the CIE L*a*b* system. The resulting parameters used to evaluate the degradation of anthocyanins in sugar solutions and water were CIE L* (lightness), CIE a* (positive values for redness; negative values for greenness) and CIE b* (positive values for yellowness; negative values for blueness). Both the SCI (Specular Component Included) and the SCE (Specular Component Excluded) modes were used for the measurements. The SCI mode was selected for the color change assessment because of the measurement of anthocyanins in glossy material, where the SCI mode accounts for this glare in the reflectance measurements. The instrument was calibrated to white (L* = 100) and black (L* = 0). The instrument parameters were mask-MAV (8 mm), gloss-SCI, UV-100% full, observer 10°, illuminant D 65 and color space-CIE L*a*b*. The data were processed using the SpectraMagic NX Lite software 1895-154 Ver.2.6 (Konica Minolta, Tokyo, Japan). The values were calculated as mean ± standard deviation for *n* = 5. The control sample was the initial model sample of anthocyanin in combination with water or sugar solution, and the resulting color change values (∆E_Lab_*) were related to this measurement as the measured initial values before the samples were placed in the thermostats.

Equation (1) was used to express the color change. For the qualitative evaluation of ∆E_Lab_*, a scale created by the authors was used [23]. See Table S1, which describes how intensely we can observe the color change.

(1) $${{∆E}^{*}}_{Lab}=\sqrt{{(L}^{*}-L_{0}^{*}{)}^{2}+{(a}^{*}-a_{0}^{*}{)}^{2}+{(b}^{*}-b_{0}^{*}{)}^{2}}$$

where L* denotes the luminance determined after some time *t*; L*_0_, the luminance value at time *t*_0_; parameters a* and b*, the values of red and blue, respectively, at time *t*; and a*_0_ with b*_0_, the values of those colors at time *t*_0_.

### 2.5. Determination of Anthocyanins by HPLC/DAD

Anthocyanins were determined through reversed-phase high-performance liquid chromatography (chromatograph Thermo Scientific™ Dionex™ UltiMate™ 3000, Thermo Scientific, Norristown, PA, USA) with gradient elution. A diode array detector (DAD) was used for detection. The measurement conditions were set according to the IFU No. 71 (1998) method “Anthocyanins by HPLC” published by the International Federation of Fruit Juice Producers. The standards cyanidin-3-*O*-*β*-glucopyranoside, cyanidin-3-*O*-*β*-galactopyranoside, cyanidin-3-*O*-*β*-rutinoside and delphinidin-3-*O*-*β*-rutinoside were used for the determination, from which calibration series of samples with concentrations were prepared (100, 50, 25, 10, 5 and 1 mg L^−1^), and the anthocyanin content of the model samples was expressed as the concentration of the respective anthocyanin that was present in the model sample. The conditions for anthocyanin measurements by HPLC-DAD were a Purospher STAR RP-8e (Merck KGaA, Darmstadt, Germany) column (5 μm), column temperature of 40 °C, mobile phase volume flow rate of 1 mL min^−1^, injection volume of 10 μL, detection at 518 nm and analysis time of 46 min. The mobile phase (MF) consisted of water/formic acid (98% p.a.) (Lach-Ner Ltd., Neratovice, Czech Republic) in a volume ratio of 9:1 (*v*/*v*) (A) and acetonitrile (>99.9%) (Chem-Lab, Zedelgem, Belgium)/water/formic acid in a volume ratio of 5:4:1 (*v*/*v*/*v*) (B), followed by a gradient of MF/isocratic 12% B for 1 min, linear gradient increase of up to 100% B over the next 34 min, leaving 100% B for 3 min, then returning to the original initial MF ratio over the next 8 min.

### 2.6. Kinetics of Anthocyanin Degradation

**Figure 1:**
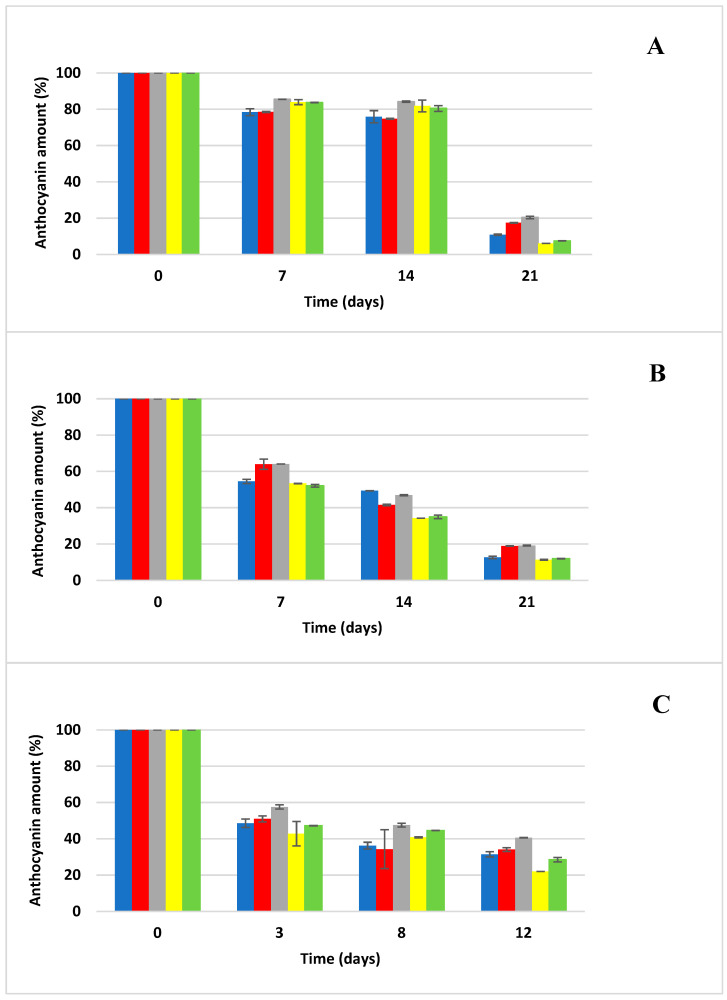
Anthocyanin cyanidin-3-*O*-*β*-glucopyranoside degradation during storage at elevated temperatures (mean ± SD); blue—distilled water, red—glucose solution, gray—sucrose solution, yellow—fructose solution, green—Glc/Fru solution; (**A**) degradation of anthocyanin at 20 °C, (**B**) degradation of anthocyanin at 35 °C, (**C**) degradation of anthocyanin at 50 °C.

**Figure 2:**
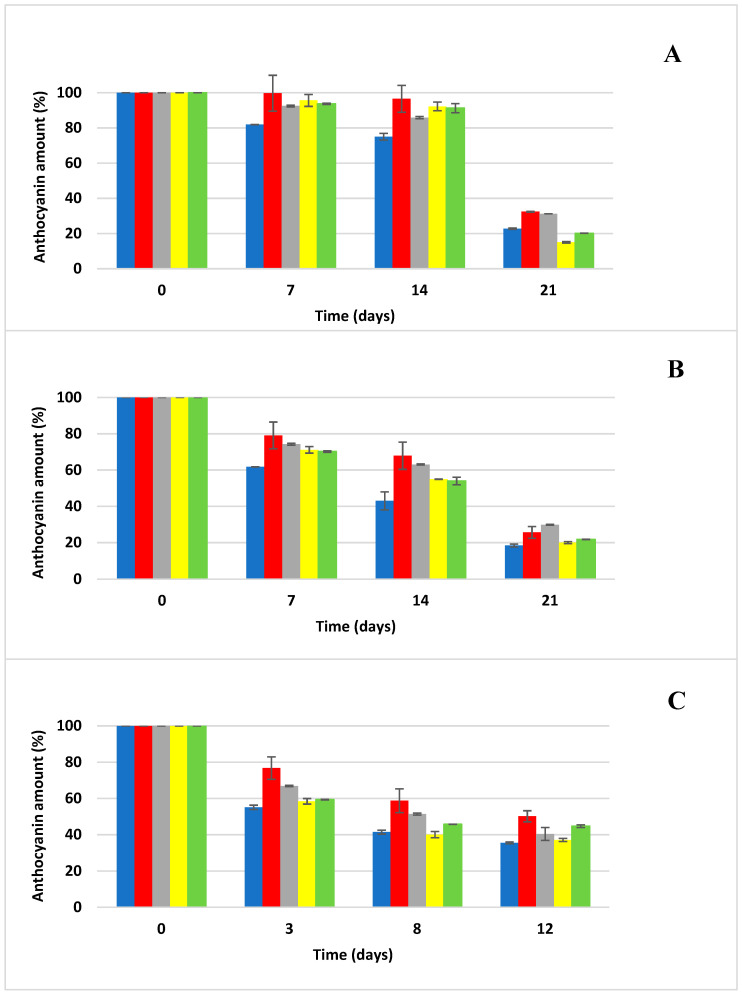
Anthocyanin cyanidin-3-*O*-*β*-rutinoside degradation during storage at elevated temperatures (mean ± SD); blue—distilled water, red—glucose solution, gray—sucrose solution, yellow—fructose solution, green—Glc/Fru solution; (**A**) degradation of anthocyanin at 20 °C, (**B**) degradation of anthocyanin at 35 °C, (**C**) degradation of anthocyanin at 50 °C.

**Figure 3:**
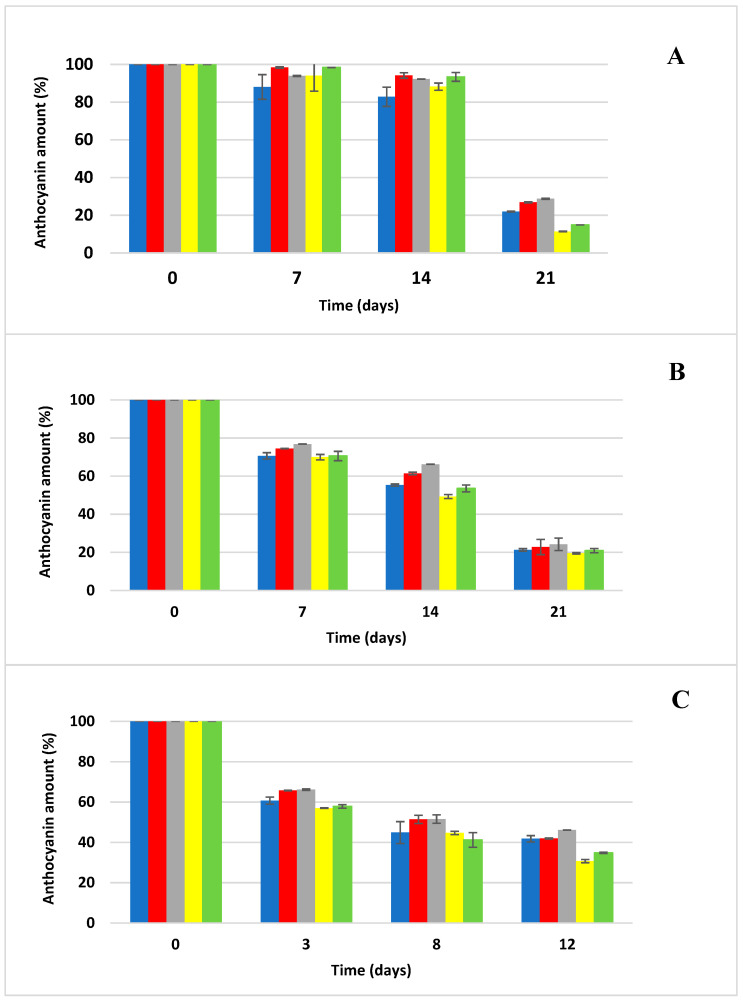
Anthocyanin cyanidin-3-*O*-*β*-galactopyranoside degradation during storage at elevated temperatures (mean ± SD); blue—distilled water, red—glucose solution, gray—sucrose solution, yellow—fructose solution, green—Glc/Fru solution; (**A**) degradation of anthocyanin at 20 °C, (**B**) degradation of anthocyanin at 35 °C, (**C**) degradation of anthocyanin at 50 °C.

**Figure 4:**
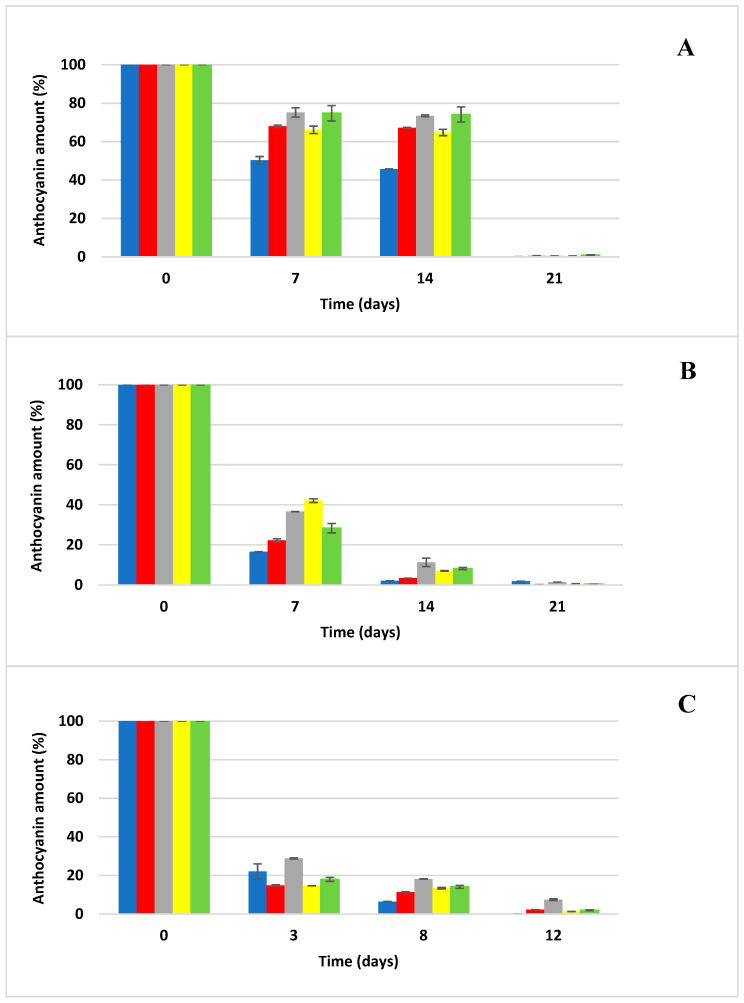
Anthocyanin delphinidin-3-*O*-*β*-rutinoside degradation during storage at elevated temperatures (mean ± SD); blue—distilled water, red—glucose solution, gray—sucrose solution, yellow—fructose solution, green—Glc/Fru solution; (**A**) degradation of anthocyanin at 20 °C, (**B**) degradation of anthocyanin at 35 °C, (**C**) degradation of anthocyanin at 50 °C.

The kinetics of anthocyanin degradation were investigated in anthocyanin samples generated for the accelerated storage test. The effect of temperature (20, 35, and 50 °C) on the degradation of anthocyanins was tested in an aqueous environment and then in four different carbohydrate solutions (glucose, fructose, sucrose and a fructose/glucose mixture (1:1)). Samples stored in thermostats (Memmert, Schwabach, Germany) at 20 °C and 35 °C were taken for the determination of anthocyanin concentration via HPLC/DAD once a week, and samples stored at 50 °C were taken twice a week because of the expected rapid degradation of anthocyanins (Figure 1, Figure 2, Figure 3 and Figure 4). The rate constants (*k*) of the first-order reaction were calculated by fitting the experimental data to the equations:

(2) $$ln\left(C_{t}/C_{0}\right)=-kt$$

where the half-life (t_1/2_) was calculated according to

(3) $$t_{1/2}=\ln\left(2\right)/k$$

where *t* and *k* denote the days after storage and rate constant (days^−1^), respectively; *C*_0_, the initial concentration of the selected anthocyanin; and *C_t_*, the concentration at time *t*.

The dependence of the rate constant of anthocyanin degradation on temperature is represented by the Arrhenius equation:

(4) $$\ln\left(k\right)=\ln\left(k_{0}\right)-E_{a}/RT$$

where *C*_0_ denotes the initial anthocyanin content; *C_t_*, the anthocyanin content after *t* minutes of heating at a given temperature; t_1/2_, the half-life; *k*, the first-order kinetic rate constant (day^−1^); *k*_0_, the frequency factor (day^−1^); *Ea*, the activation energy (kJ moL^−1^); *R*, the universal gas constant (8.314 J moL^−1^ K^−1^); and *T*, the absolute temperature (*K*). The degradation kinetics of anthocyanins were determined based on the procedures applied by the researchers in their studies [24,25].

### 2.7. Statistical Analysis

Statistica 14.0 software (StatSoft, Prague, Czech Republic) was used for the statistical evaluation of the data. To verify the normality of the resulting data, the data were subjected to graphical and analytical methods. In the Statistica software, the normality of the data distribution was confirmed graphically using a histogram and then analytically using the Shapiro–Wilk test. The statistical significance level for the resulting values was set at *p* < 0.05. To test for statistically significant differences regarding the effect of the different water activities of sugar solutions on the retardation of anthocyanin degradation, *t*-tests were conducted. A multivariate ANOVA (MANOVA) with multivariate Hotelling’s test was conducted to test for statistically significant differences between the resulting degradation of anthocyanins as affected by storage temperature, the use of different carbohydrate types and the form of glycosidation of the selected aglycone. A post-hoc Tukey HSD test was conducted to find statistically significant differences between the selected anthocyanins and the carbohydrates used. Linear regression was employed to determine the rate constants of anthocyanin degradation using Microsoft Excel. HPLC analysis was conducted in triplicate, and the results were expressed as mean ± standard deviation. The spectrophotometric parameters for the determination of anthocyanin color in the CIEL*a*b* space were determined five times for each sample (L*, a*, b*). Each test was carried out in duplicate. Spearman’s correlation coefficient (ρ) was determined between parameter a* and anthocyanin concentration loss as well as for the correlation of the temperature dependence of anthocyanins and parameter a* to the rate constant (*k*) using the Statistica software.

## 3. Results and Discussion

### 3.1. Verification of the HPLC/DAD Method for the Determination of Anthocyanins

A conventional HPLC/DAD method was selected for the determination of anthocyanin concentration using the IFU No. 71 method published by the International Federation of Fruit Juice Producers (1998). The parameters limit of detection (LOD), limit of quantification (LOQ) as well as linearity and repeatability were determined for each anthocyanin used in the sucrose environment as the conditions are expected to be extremely similar in the other carbohydrates used. Repeatability was expressed as relative standard deviation (RSD = %) for n = 5. The selected chromatograms of the anthocyanin standards are given in the Supplementary Materials Section (Figures S1 and S2). The calibration curves of all four anthocyanin standards were utilized to evaluate the content of each anthocyanin. To evaluate the resulting Cya3Glc concentration, a calibration line equation of the form mAU = 0.2302c + 0.3535 with a coefficient of determination with R^2^ = 0.9946 was used, and linearity was determined from 2.13 to 100 mg L^−1^. The LOD and LOQ for a given anthocyanin were calculated as 3 and 10 times the noise height to the calibration line directive to 0.65 and 2.17 mg L^−1^, respectively. The repeatability expressed as relative standard deviation (n = 5) was RSD = 1.21%. For Cya3Rut, the LOD and LOQ were calculated to be 0.74 and 2.45 mg L^−1^, respectively, and the linearity of the measurement range was from 2.45 to 100 mg L^−1^. The equation of the calibration curve used to quantify the resulting concentrations was mAU = 0.2652c + 0.275 with R^2^ = 0.9509, and the repeatability was RSD = 0.21%. The validation parameters for Cya3Gal as the linearity of the range of determination was determined from 1.20 to 100 mg L^−1^; the LOD and LOQ were calculated to be 0.36 and 1.20 mg L^−1^, respectively; and the RSD value was 0.19%. The calibration equation was mAU = 0.375c + 0.394 with R^2^ = 0.9864. For the last anthocyanin standard Del3Rut, the LOD and LOQ parameters were 1.01 and 3.36 mg L^−1^, respectively, and the linearity of the method of determination was from 3.36 to 100 mg L^−1^, whereas the repeatability was calculated to be RSD = 2.07%. The equation of the calibration curve for the determination of the final concentration of Del3Rut in the samples was mAU = 0.149c + 0.2281. Confirmation of the determined anthocyanins in the samples was conducted on the basis of external calibration and then according to the recorded absorption spectra within the HPLC method, which were in agreement with the available literature [26,27].

### 3.2. Percentage Loss of Anthocyanins During Storage at Elevated Temperatures

Model samples of anthocyanins (cyanidin-3-*O*-*β*-glucopyranoside, cyanidin-3-*O*-*β*-galactopyranoside, cyanidin-3-*O*-*β*-rutinoside and delphinidin-3-*O*-*β*-rutinoside) were prepared in the presence of selected sugar solutions (sucrose, glucose, fructose and a mixture of glucose and fructose in a ratio of 1:1 (*w*/*w*)) for the accelerated storage test, in which the standard conditions were set for the final model samples and monitored during 4 weeks of storage at elevated temperatures (20, 35, and 50 °C). The three selected temperatures were chosen on the basis of information on the thermolability of anthocyanins. The aforementioned anthocyanins were selected owing to their predominance in fruits and vegetables [28]. The pH of all the samples was maintained at 3.4 using acetate buffer due to the predominantly colored form of anthocyanins in the form of the flavylium cation and their increased stability [4,29,30]; water activity was monitored at 25 °C, which was reduced by using 25% sugar solutions to values ranging from 0.9691 ± 0.0016 for glucose, 0.9708 ± 0.0009 for the Fru/Glc mixture and 0.9741 ± 0.0008 to 0.9843 ± 0.0005 for sucrose. Using *t*-test, it was confirmed that the above difference within the change in water activity of sugar solutions did not have a statistically significant effect on the retardation of anthocyanin degradation.

The degradation of all anthocyanin standards was monitored over a period of 4 weeks (the graphs and tables show determinations for 3 weeks), and no residual anthocyanins were determined in the model samples after this storage period. Anthocyanins stored at lower temperatures (20 °C and 35 °C) were still determined in the model samples after 3 weeks of storage, but at 50 °C, the anthocyanins were completely degraded in 14 days of storage. A study made by Liu et al. [31] reported a 50% reduction in the total anthocyanin content (TAC) of blueberries during 72 h of storage at 50 °C, which could be due to some interfering substances, such as ascorbic acid, which exerts a positive effect on slowing down the degradation of anthocyanins [4,32].

It can be seen from Figure 2 that Cya3Rut shows higher stability than Cya3Glc during the accelerated storage test at all the storage temperatures used (20, 35, and 50 °C). However, Figure 4 shows that glycosidation with rutinoside does not affect the higher stability of the selected anthocyanins, as Del3Rut degraded significantly faster than the rest of the selected anthocyanin standards within all the storage temperatures used. A study that explored the degradation of a similar range of selected anthocyanins as in our experiment, but in different types of fruit juices, reported that delphinidin degraded the fastest at 21 °C among all the selected anthocyanins, which is consistent with our model samples consisting of an acidified solution of selected carbohydrates fortified with an anthocyanin standard [33].

The experimental conditions were standardized with respect to the pH of the model samples, the water activity of the sugar solutions and the initial anthocyanin concentrations, factors that undoubtedly influence the final degradation kinetics of the anthocyanins used [9,12,20]. As can be seen from Figure 1, Figure 2, Figure 3 and Figure 4, sucrose (25% solution in H_2_O) exerted the most protective effect on the anthocyanins used, significantly (*p* < 0.05) retarding the degradation of the selected anthocyanin standards across all storage temperatures. On the other hand, at low sucrose concentrations (86 g L^−1^), the degradation of anthocyanins from extracts of red cabbage, blackcurrant and elderberry was higher in soft drinks compared to buffered systems at pH = 3, while the opposite was observed for grape extract [34]. In another study, the stability of anthocyanins in extracts of grape marc, elderberry and blackcurrant was lower in all systems with added sucrose (100 g L^−1^) compared to control samples at pH = 3, 4 and 5, while the browning index did not change with added sugar [35]. Adding 20 g L^−1^ of sucrose to a model drink (pH = 3) containing red cabbage and grape extracts did not affect the thermal stability and photostability of anthocyanins [36]. Although most extracts of anthocyanins showed lower stability in systems with added sugar, no statistical analysis was conducted to verify the significance of this difference. In a scientific publication conducted by Rosso et al. [10], they stated that the addition of sugars and salts had a negative effect on the stability of anthocyanins from both natural sources (açai and acerola) and that systems with added açai were significantly more stable than those with added acerola; these results show that the stability of the addition of natural anthocyanin extract to soft drinks depends strongly on the composition of anthocyanins from the natural material used. Meanwhile, the use of fructose solution significantly accelerated the degradation of anthocyanins, and the degradation even progressed faster than when anthocyanins were stored in distilled water alone. This was confirmed in the study by Cao et al. [11] when they reported the significant negative effect of fructose on slowing down the degradation of anthocyanins. The glucose solution even showed a higher protective effect on retarding degradation in the case of Cya3Rut storage, but the result was not statistically significant. The glucose solution also exerted similar protective effects on the sucrose solution in other cases of storage of the remaining anthocyanins. Cya3Rut was the most stable anthocyanin, but Cya3Gal also exhibited an extremely similar stability during storage at 50 °C. In a study by Hou et al. [26], Cya3Rut also proved to be the most stable among the selected anthocyanins at temperatures of up to 90 °C.

#### Correlation of Parameters a* and b* with Anthocyanin Concentration Loss

In an accelerated storage test with model anthocyanin samples in the presence of sugar solutions, the concentration of each anthocyanin was determined after a period of days of storage. Before the actual HPLC analysis of the model samples, spectrophotometric determination of the L*, a* and b* values was conducted. Specifically, a* was selected as the key parameter for the qualitative assessment of anthocyanin color degradation. From the resulting values of the a* parameter (n = 5) and the determined anthocyanin concentration, the dependencies are plotted in Figure 1, Figure 2, Figure 3 and Figure 4, with the red color degradation of anthocyanins correlated with the loss (%) of the selected anthocyanins shown on the minor axis of the plots. Spearman’s correlation coefficients were calculated for each anthocyanin using the Statistica 14.0 software. The correlations were significant at the *p* < 0.05 level, and the value of the correlation coefficient with parameter a* was ρ = 0.6713 for Cya3Glc, which is very abundant in raspberries [37] but also in blackcurrants [38]. The correlation with the b* parameter was calculated with a value of ρ = −0.7441. For Cya3Rut, Spearman’s correlation coefficient had a value of ρ = 0.5917 in relation to red color degradation. Cya3Rut is abundant in mulberry [39] and in black raspberry [40]; therefore, the correlation value with parameter b* was ρ = −0.6870 due to the color of the crop in question. Cya3Gal exhibited a significant correlation with the value of ρ = 0.7207 but had the lowest correlation (*p* < 0.05) with the b* parameter of all the anthocyanins selected, with a value of ρ = 0.3590. This could have been due to the different color spectrum of Cya3Gal, where its sources mainly included chokeberry [41]. The last of the anthocyanins, Del3Rut, was correlated with parameter a* with a value of ρ = 0.6963. For Del3Rut, the correlation with parameter b* was calculated due to the original color of the anthocyanin standard, which was initially darker than the other cyanidins. It is the very high Del3Rut content found, for example, in the skin of aubergine [42] and especially in blackcurrants [43]. The value of the correlation coefficient was ρ = −0.8089. From the resulting correlations, it can be assumed that the rapid spectrophotometric determination of the CIEL*a*b* parameters can predict the decrease in anthocyanin concentration in the samples. This fact was confirmed in a study conducted by Rampáčková et al. [44], who determined the color parameters CIEL*a*b* using an identical colorimeter (Konica Minolta, Tokyo, Japan) and defined the significant negative correlations of the parameters L* and b* with the change in TAC but in the samples of dark plums. Due to the very similar value of the L* parameter (Table 1 for all model anthocyanin samples, no significant correlation with anthocyanin loss was observed in this experiment.

**Table 1:** Determined parameters by spectrophotometric method CIEL*a*b* (Konica Minolta, Japan) and evaluated color change of anthocyanins.

Anthocyanins	Solvent	Temperature (°C)	L*	a*	b*	∆E *_Lab_
Cya3Glc ^1^	Water	20	23.78 ± 0.01	1.64 ± 0.02	−1.89 ± 0.02	1.35 ± 0.02
		35	23.83 ± 0.01	1.40 ± 0.03	−1.12 ± 0.01	1.85 ± 0.03
		50	24.25 ± 0.01	1.36 ± 0.00	−0.71 ± 0.02	2.59 ± 0.02
	Glucose	20	23.47 ± 0.01	1.59 ± 0.02	−2.34 ^b,c^ ± 0.02	2.30 ± 0.02
		35	24.12 ± 0.01	1.65 ± 0.01	−0.92 ± 0.01	2.22 ± 0.02
		50	23.79 ± 0.01	1.01 ± 0.01	−0.53 ± 0.01	2.95 ± 0.02
	Sucrose	20	23.57 ± 0.01	2.24 ^c^ ± 0.02	−1.42 ± 0.02	0.45 ± 0.03
		35	24.36 ± 0.01	2.11 ± 0.01	−1.10 ± 0.01	1.00 ± 0.02
		50	24.21 ± 0.02	1.80 ^a^ ± 0.03	−0.95 ± 0.02	1.18 ± 0.03
	Fructose	20	23.68 ± 0.01	2.16 ± 0.02	−1.37 ^b,c^ ± 0.02	0.77 ± 0.03
		35	24.18 ± 0.00	2.34 ± 0.01	−0.66 ^a^ ± 0.02	1.26 ± 0.02
		50	24.06 ± 0.00	2.56 ± 0.06	−0.48 ^a^ ± 0.02	2.37 ± 0.06
	Fructose/glucose	20	23.43 ± 0.01	1.72 ± 0.02	−1.54 ± 0.02	0.83 ± 0.02
		35	24.28 ± 0.01	1.95 ± 0.02	−0.58 ± 0.02	1.34 ± 0.02
		50	24.41 ± 0.01	1.89 ± 0.02	−0.46 ± 0.02	1.51 ± 0.01
Cya3Rut ^2^	Water	20	24.20 ± 0.01	1.89 ± 0.03	−1.67 ± 0.01	1.29 ± 0.03
		35	24.47 ± 0.01	1.57 ± 0.02	−0.75 ± 0.01	1.79 ± 0.02
		50	24.67 ± 0.01	2.00 ± 0.01	−0.38 ± 0.01	1.70 ± 0.01
	Glucose	20	23.62 ± 0.00	1.85 ± 0.03	−1.60 ± 0.01	0.35 ± 0.03
		35	24.25 ± 0.01	1.82 ± 0.03	−0.77 ± 0.02	1.05 ± 0.03
		50	24.31 ± 0.01	1.57 ± 0.03	−0.48 ± 0.02	1.41 ± 0.02
	Sucrose	20	23.96 ± 0.01	2.43 ^b,c^ ± 0.02	−1.73 ^b,c^ ± 0.02	0.52 ± 0.02
		35	24.03 ± 0.02	1.87 ^a^ ± 0.02	−0.87 ^a^ ± 0.02	0.93 ± 0.03
		50	23.74 ± 0.01	1.51 ^a^ ± 0.02	−0.48 ^a^ ± 0.02	1.38 ± 0.02
	Fructose	20	23.66 ± 0.01	2.48 ± 0.02	−1.21 ^c^ ± 0.02	0.47 ± 0.02
		35	24.34 ± 0.02	2.76 ± 0.02	−0.84 ± 0.02	0.99 ± 0.02
		50	24.48 ± 0.01	2.65 ± 0.02	−0.40 ^a^ ± 0.00	1.39 ± 0.01
	Fructose/glucose	20	23.75 ± 0.01	2.26 ± 0.02	−1.40 ± 0.01	0.60 ± 0.02
		35	24.32 ± 0.01	2.25 ± 0.02	−0.77 ± 0.01	1.04 ± 0.01
		50	24.47 ± 0.00	2.30 ± 0.02	−0.63 ± 0.02	1.18 ± 0.01
Cya3Gal ^3^	Water	20	24.38 ± 0.01	3.15 ± 0.02	−0.78 ± 0.02	2.69 ± 0.01
		35	24.80 ± 0.01	2.49 ± 0.01	−0.58 ± 0.02	3.02 ± 0.02
		50	24.62 ± 0.01	3.05 ± 0.02	−0.01 ± 0.02	2.32 ± 0.01
	Glucose	20	24.00 ± 0.01	3.63 ± 0.01	−0.68 ± 0.01	2.09 ± 0.01
		35	24.40 ± 0.01	3.32 ± 0.02	−0.28 ± 0.01	1.99 ± 0.01
		50	24.45 ± 0.01	2.18 ± 0.01	−0.53 ± 0.01	3.10 ± 0.01
	Sucrose	20	24.03 ± 0.01	3.40 ^c^ ± 0.03	−0.59 ± 0.02	1.86 ± 0.03
		35	24.18 ± 0.00	3.05 ^c^ ± 0.01	−0.44 ± 0.01	1.95 ± 0.01
		50	23.80 ± 0.01	1.72 ^a,b^ ± 0.01	−0.57 ± 0.01	3.21 ± 0.01
	Fructose	20	23.90 ± 0.01	2.63 ± 0.03	−0.40 ^c^ ± 0.01	3.67 ± 0.03
		35	24.20 ± 0.01	3.50 ± 0.02	−0.18 ± 0.01	2.76 ± 0.03
		50	24.50 ± 0.00	3.14 ± 0.03	0.28 ^a^ ± 0.02	2.83 ± 0.03
	Fructose/glucose	20	23.69 ± 0.01	2.64 ± 0.01	−0.51 ± 0.05	0.78 ± 0.02
		35	24.28 ± 0.00	2.54 ± 0.04	−0.53 ± 0.01	0.67 ± 0.02
		50	24.31 ± 0.01	2.79 ± 0.03	−0.04 ± 0.02	2.05 ± 0.03
Del3Rut ^4^	Water	20	25.40 ± 0.01	0.39 ± 0.02	0.64 ^b^ ± 0.02	3.93 ± 0.01
		35	24.49 ± 0.00	0.14 ± 0.01	−0.10 ^a,c^ ± 0.01	3.05 ± 0.01
		50	25.30 ± 0.01	0.28 ± 0.01	0.31 ^b^ ± 0.01	3.63 ± 0.02
	Glucose	20	25.42 ± 0.01	0.60 ^c^ ± 0.02	0.79 ± 0.01	4.08 ± 0.03
		35	24.56 ± 0.01	0.56 ± 0.01	0.01 ± 0.01	2.98 ± 0.01
		50	24.49 ± 0.01	0.23 ^a^ ± 0.01	−0.33 ± 0.02	2.70 ± 0.01
	Sucrose	20	25.17 ± 0.01	0.46 ± 0.02	0.71 ^b,c^ ± 0.01	3.70 ± 0.02
		35	24.13 ± 0.01	0.21 ± 0.02	−0.33 ^a^ ± 0.01	2.40 ± 0.01
		50	24.89 ± 0.01	0.67 ± 0.00	−0.23 ^a^ ± 0.01	2.74 ± 0.01
	Fructose	20	25.77 ± 0.01	0.76 ^b,c^ ± 0.01	1.49 ^b,c^ ± 0.02	4.63 ± 0.01
		35	25.20 ± 0.00	0.55 ^a^ ± 0.03	0.59 ^a^ ± 0.03	3.60 ± 0.00
		50	25.42 ± 0.01	0.56 ^a^ ± 0.01	0.76 ^a^ ± 0.01	3.84 ± 0.02
	Fructose/glucose	20	25.89 ± 0.00	0.50 ± 0.01	1.15 ^b,c^ ± 0.02	4.53 ± 0.01
		35	24.88 ± 0.00	0.31 ± 0.01	0.14 ^a^ ± 0.01	3.23 ± 0.00
		50	25.18 ± 0.00	0.84 ± 0.01	0.48 ^a^ ± 0.02	3.66 ± 0.02

^1^ cyanidin-3-O-glucoside; ^2^ cyanidin-3-O-rutinoside; ^3^ cyanidin-3-O-galactoside; ^4^ delphinidin-3-O-rutinoside; resulting values of individual parameters are presented as Mean ± SD (n = 5). a–c upper indexes—means in the same temperature line followed by different indexes represent significant differences.

### 3.3. Determination of Selected CIELab Parameters (L*, a*, b*) and Color Change (∆E_Lab_ *) of Anthocyanins During Accelerated Storage Test

Before the actual determination of anthocyanin concentration through HPLC/DAD, the L*, a* and b* parameters for each model anthocyanin sample were determined in the presence of the carbohydrates and water used. From these parameters, the total color change (∆E_Lab_*) was calculated using Equation (1); the resulting data for all samples are shown in Table 1. The color lightness (L*) of the model anthocyanin samples did not significantly change during storage, as can be seen from the values in Table 1. An extremely similar value for the lightness of the samples was determined in a study by Loypimai et al. [24]. The overall color change was very clear according to the recommended rating scale for this parameter proposed by Shevell with slightly modifications [23]. Table S1 shows that for most samples, the change was obvious at first glance or in some cases revealed on closer examination. The overall color change significantly varied with increasing storage temperature for all model anthocyanin samples. However, most of the studies have been conducted on real food matrix samples, such as plums [44], sweet potatoes [45], beetroot [46] or raspberries [47]. When the selected anthocyanins Cya3Glc and Cya3Rut were stored at 20 °C in the presence of sucrose, the overall color change was minimal from the beginning of storage. This could be due to the protective effect of sucrose on the degradation of anthocyanins at elevated storage temperatures. The b* parameter decreased from blue color towards yellow during storage. This could be due to the formation of degradation products of anthocyanins but also to the carbohydrates present in the model samples, which are formed during storage at elevated temperatures and in acidic environments. From the values of the chromatic parameters, it can be observed that the most stable anthocyanin was Cya3Rut, whose ∆E_Lab_* value did not exceed 2.0 in any case. From the resulting values for the ∆E_Lab_* parameter, it is clear that the greatest changes within the selected anthocyanins occurred in samples containing Del3Rut, which exhibited the greatest changes in spectrophotometric color determination. Table 1 shows that the results determined for individual anthocyanins in the sugar solution environment, namely sucrose and fructose, were significant in the statistical analysis. Temperature was considered as a variable factor for the determination of statistically significant results in the color determination, since after the initial static evaluation of the results using MANOVA analysis, temperature was found to be the statistically significant factor most influencing the degradation rate of individual anthocyanins. Temperature has been confirmed to play a major role in color changes and gradual degradation of anthocyanin content in model samples.

### 3.4. Degradation Kinetics of Anthocyanins at Elevated Storage Temperatures Involving Different Carbohydrates

The parameters of the degradation kinetics of the model samples of anthocyanins were calculated. Based on published studies on anthocyanin degradation kinetics, the first-order kinetic reaction of anthocyanin degradation during storage at elevated temperatures (20, 35, and 50 °C) was determined and confirmed [25,26]. The rate constants (*k*) of the first-order reaction were estimated by fitting the experimental data to Equation (2) and the half-lives of the selected anthocyanins to Equation (3).

The kinetic parameters (*k*, *t*_1/2_) for the model samples of anthocyanins in the presence of sugar solutions during storage at elevated temperatures are presented in Table 2. From the resultant anthocyanin degradation kinetics data, it is evident that with the elevated storage temperature of the model samples, the value of the kinetic reaction rate constant also increases and the half-life of the selected anthocyanins decreases. The samples of individual anthocyanins in the presence of the carbohydrates Glc, Fru and Glc/Fru were not statistically significantly different from the model samples of anthocyanins with water. Even the samples of single anthocyanins stored in distilled water (pH = 3.4) at elevated temperatures exhibited higher stability than the samples of anthocyanins in the presence of Fru. The negative effect of Fru on the degradation rate of anthocyanins was reported by Cao et al. [11]. The negative effect on slowing down the degradation of anthocyanins was demonstrated (*p* < 0.05) specifically for samples containing sucrose solution, where substantial differences were observed, especially when the model anthocyanin samples were stored at higher temperatures (35 °C and 50 °C) with coefficient of determination values ranging from R^2^ = 0.8381 to R^2^ = 0.9844. The coefficient of determination values for samples stored at 20 °C ranged from R^2^ = 0.6229 to R^2^ = 0.7617. The lower precision of the determinations could be due to the slightly different behavior of the anthocyanins during storage. A study by Roidoung et al. [47] reported kinetic modelling of anthocyanin degradation, outlining the order of magnitude of the kinetic reaction from *n* = 1.2 to 4.4. For example, Yang et al. [46] reported that the anthocyanins extracted from beet have no simple trend in the degradation kinetics. Within the degradation kinetics of anthocyanins, most studies reported that the degradation pathway was governed by first-order kinetics [26,48,49].

According to the resulting data, the least-stable model sample of anthocyanin was Del3Rut, where the half-life of the anthocyanin ranged from 2.4 to 7 days from the start of storage in individual thermostats. A study by Hellström et al. [33] also investigated the degradation of Del3Rut at 21 °C but in blackcurrant juice samples. The half-life of Del3Rut in their study was calculated to be 2.71 weeks, which is quite significantly longer than in our case. This phenomenon could be due to the substantial co-pigmentation [49] of anthocyanins in food matrices, where anthocyanins are stabilized by various associated substances (acylation, glycosidation of anthocyanins).

The resulting rate constants were statistically significantly different across the different storage temperatures. Statistically significant differences were observed between the storage temperatures of 20, 35, and 50 °C through ANOVA and a subsequent post hoc Tukey HSD test. No statistically significant differences were observed in the stability of the stored model anthocyanin samples in the presence of the selected carbohydrates between 35 °C and 50 °C.

The resulting data also indicate that the Glc/Fru mixture does not have the same protective effect as sucrose, which exerts in all cases the best protective effect on slowing down the degradation of anthocyanins in the model samples. This could be due to the non-reducing properties of sucrose (lower reactivity against reducing monosaccharides), which has been demonstrated to retard the degradation of the selected anthocyanins in the model samples. Tsai et al. [22] even mentioned that the addition of sucrose enhances the antioxidant capacity of mulberry anthocyanin extract. Maillard reactions were reported in several scientific papers, which caused faster degradation of anthocyanins in various food matrices in the presence of added carbohydrates at elevated storage temperatures [50,51,52]. In our experiment, these reactions were eliminated by the preparation of model samples, in which no real food matrix was involved, where there was a possibility of reactions between amino compounds and carbohydrates, and thus, no consideration was given to acceleration of the degradation of the selected anthocyanins due to the resulting undesirable by-products of these reactions. Moreover, in our experiment, the storage temperatures ranged from 20 °C to 50 °C, a range in which such reactions are quite slow compared with storage temperatures above 50 °C. The accelerated degradation of anthocyanins in the presence of fructose may have been mainly due to the formation of carbohydrate degradation products in an acidic environment. It has been reported that 5-HMF (5-hydroxymethylfurfural) accelerates the degradation of anthocyanins [51]. Lee and Nagy [52] even reported that fructose in acidic environments produces 5-HMF most rapidly among all the monosaccharides used in our experiment. This could explain that fructose, in turn, exerts an accelerating effect on anthocyanin degradation. Most scientific studies [11,25,47] focusing on the effect of carbohydrates on the potential retardation of anthocyanin degradation at elevated temperatures have been conducted on real food matrices. A co-pigmentation in this case was involved in the processes of potential retardation of the degradation of the analytes of interest, possibly biasing some of the results in the observation of the effectiveness of carbohydrates on retarding the degradation of some anthocyanins. In our study, the experiment was conducted with pure substances, which eliminated the influence of some potential retarders of anthocyanin degradation, thus yielding extremely robust results.

#### Temperature Dependence of Anthocyanins and Parameter a* and Their Correlation

The dependence of the degradation kinetic constant on temperature was represented by a relation based on the Arrhenius equation (Equation (4)). The graphs for the degradation rate of individual anthocyanins at different storage temperatures were constructed and are presented in Supplementary Materials (Figures S3–S6). A similar approach to the evaluation of the kinetic parameter (E_a_) was employed in some previous studies [24,26,53]. Ahmed et al. [54] reported that the higher the activation energy value, the higher the temperature sensitivity of the substance of interest. They also concluded that anthocyanins are more sensitive to temperature than parameter a*, which is not in agreement with the results presented in Table 3. The resulting data indicated that parameter a* is extremely sensitive to temperature change during storage as the value of E_a_ was substantially higher than that of anthocyanins alone. The kinetic parameters of the a* value could partially predict the degradation of anthocyanins in the accelerated storage test. It can be seen from Table 3 that the resulting values were accurate with the value of the coefficient of determination for Del3Rut ranging from 0.8534 < R^2^ < 0.9992, and for parameter a*, the value was from 0.7686 < R^2^ < 0.9142. Moreover, for Cya3Glc, the values ranged from 0.7844 < R^2^ < 0.9946, and for parameter a*, the values ranged from 0.7883 < R^2^ < 0.9792. For Cya3Rut, the values ranged from 0.7775 < R^2^ < 0.9688, and for parameter a*, the values ranged from 0.8294 < R^2^ < 0.9802. For the last anthocyanin, Cya3Gal, the values of the coefficient ranged from 0.7965 < R^2^ < 0.9981, and for parameter a*, the values ranged from 0.8139 < R^2^ < 0.9829.

From the calculated values of activation energies for the selected anthocyanins in the presence of different carbohydrates, it is clear that Del3Rut stored in water was the most stable and most sensitive to temperature change with a value of E_a_ = 27.27 kJ moL^−1^. Meanwhile, the highest value of activation energy in terms of the change in parameter a* was observed for Cya3Rut stored in the presence of fructose with a value of E_a_ = 68.02 kJ moL^−1^. This fact is consistent with the statement that fructose did not exert a protective effect on anthocyanins against degradation and, on the contrary, accelerated the reaction upon temperature change. Cya3Rut was the least stable in the environment of the Fru/Glc mixture with E_a_ = 0.87 kJ moL^−1^. It can be further observed from the data that in the determination of this kinetic parameter (E_a_), the environment in which the anthocyanins were stored did not affect their stability. From the resulting E_a_ for parameter a*, it can be concluded (Table 3) that carbohydrates exert a protective effect compared with aqueous environments, but this is not the case for the resulting E_a_ values for anthocyanins. Table 3 shows that the data for the resulting activation energies calculated for anthocyanins and the a* parameter are very well correlated. For all anthocyanin model samples, the correlation was determined using Spearman’s correlation coefficient at the *p* < 0.05 level of statistical significance, with values specifically for Del3Rut in aqueous media of ρ = 0.9419; for Suc, ρ = 0.7310; for Glc, ρ = 0.6257; for Fru, ρ = 0.7399; and for the Fru/Glc mixture, ρ = 0.9642. The resulting correlation coefficient values for Cya3Gal were as follows: ρ = 0.6804 (sucrose) < ρ = 0.7952 (glucose) < ρ = 0.9710 (fructose) < ρ = 0.9863 (water) < ρ = 0.9975 (fructose/glucose). For Cya3Rut, the following values were ρ = 0.7679 (fructose) < ρ = 0.8909 (glucose) < ρ = 0.9653 (fructose/glucose) < ρ = 0.9655 (sucrose) < ρ = 0.9694 (water). The last anthocyanin selected was Cya3Glc, with values of ρ = 0.7617 (water) < ρ = 0.9434 (fructose/glucose) < ρ = 0.9619 (sucrose) < ρ = 0.9901 (fructose) < ρ = 0.9999 (glucose). The highest value of correlation between parameter a* and anthocyanin degradation was observed for the model sample Cya3Glc in the presence of glucose, whereas the lowest value of correlation was determined for the sample Del3Rut also in the presence of glucose.

Another aim of this study was to determine the existence of a difference in the degree of protective effect against anthocyanin degradation in the case of glycosidation by a monosaccharide or oligosaccharide unit. In this study, the potential higher protective effect on degradation retardation during storage at elevated temperatures of those anthocyanins glycosylated with an oligosaccharide (rutinose) versus a monosaccharide (glucose, galactose) was considered. The effect of the acylation and glycosidation of hydroxycinnamic acid bound to anthocyanin and their influence on the stability of anthocyanins under accelerated storage test was reported by De Marchi [25]. Their study reported that anthocyanins derived from red cabbage are stabilized by the acylated form of hydroxycinnamic acid, which is bound to the corresponding anthocyanin.

## 4. Conclusions

The aim of this research was to verify and determine the suitability of selected carbohydrates as protective agents for anthocyanins based on the assessment of color changes during an accelerated storage experiment and the kinetics of the degradation reaction of selected anthocyanin solutions.

The model samples of four different anthocyanins (cyanidin-3-*O*-*β*-glucopyranoside, cyanidin-3-*O*-*β*-galactopyranoside, cyanidin-3-*O*-*β*-rutinoside and delphinidin-3-*O*-*β*-rutinoside) were prepared in the presence of water and four different sugar solutions (glucose, sucrose, fructose, and fructose/glucose mixture) (25% (*w*/*w*)) adjusted to pH = 3.4 with acetate buffer and a uniform anthocyanin concentration (0.15 mol L^−1^) at the beginning of the experiment. An accelerated storage test was conducted for 4 weeks at 20, 35, and 50 °C, and the concentration of individual anthocyanins was determined after certain intervals through HPLC/DAD and spectrophotometric parameters CIEL*a*b*. The kinetic parameters (*k*, t_1/2_, E_a_) describing the degradation of anthocyanins as well as the red color change (a*) were determined in the experiment. First-order degradation kinetics were confirmed for all the model samples of anthocyanins in the presence of water and sugar solutions. Significant correlation coefficients were determined between the rate constants of anthocyanin degradation and the red color degradation (a*), which were also determined for the change in anthocyanin concentration during the accelerated storage test and red color degradation (a*) during storage at elevated temperatures. It was found that the selected anthocyanins degraded more slowly in the presence of sucrose at elevated temperatures than in the presence of the other carbohydrates and water used. Simultaneously, the effect of the different glycosidation of the anthocyanin aglycone was not statistically significant, with no evidence of higher stability of rutinosides within the selected anthocyanins. Fructose was confirmed to accelerate anthocyanin degradation during storage at elevated temperatures. From the resulting data, it was determined that the stability of the selected anthocyanins decreased in the order cyanidin-3-*O*-*β*-rutinoside > cyanidin-3-*O*-*β*-galactopyranoside > cyanidin-3-*O*-*β*-glucopyranoside > delphinidin-3-*O*-*β*-rutinoside.

By determining the correlation between anthocyanin degradation and red a*, the possibility of partial prediction of anthocyanin degradation was presented. This could be applicable in the food industry as a rapid method for the approximate determination of anthocyanin loss in samples.

## Figures and Tables

**Table 2:** Effect of temperature and selected sugar solution on the parameters of anthocyanin degradation kinetics during storage.

Solvent	Temperature (°C)	Cya3Glc ^a^		Cya3Rut ^b^		Cya3Gal ^c^		Del3Rut ^d^	
		*k* ^f^ (Days)^−1^	*t*_1/2_ ^g^ (Days)	*k* (Days)^−1^	*t*_1/2_ (Days)	*k* (Days)^−1^	*t*_1/2_ (Days)	*k* (Days)^−1^	*t*_1/2_ (Days)
Water	20	0.632 (0.7023) ^e^	7.7	0.453 (0.7617)	10.7	0.461 (0.7030)	10.5	1.415 (0.6885)	6.9
	35	0.658 (0.8690)	7.4	0.544 (0.9672)	8.9	0.489 (0.9047)	9.9	2.197 (0.8976)	4.4
	50	0.668 (0.8893)	7.3	0.593 (0.9193)	8.2	0.512 (0.9012)	9.5	4.014 (0.9026)	2.4
Glucose	20	0.529 (0.7384)	9.2	0.341 (0.6229)	14.2	0.399 (0.6372)	12.2	1.613 (0.6605)	6.0
	35	0.544 (0.9764)	8.9	0.413 (0.8301)	11.8	0.468 (0.8664)	10.4	2.047 (0.9739)	4.7
	50	0.644 (0.8364)	7.5	0.430 (0.9899)	11.3	0.500 (0.9703)	9.7	2.110 (0.9357)	4.6
Sucrose	20	0.479 (0.6888)	10.1	0.357 (0.7083)	13.6	0.376 (0.6531)	12.9	1.395 (0.6464)	7.0
	35	0.507 (0.9478)	9.6	0.379 (0.9039)	12.8	0.444 (0.8381)	10.9	1.446 (0.9703)	6.7
	50	0.528 (0.9043)	9.2	0.523 (0.9844)	9.3	0.450 (0.9355)	10.8	1.627 (0.9704)	6.0
Fructose	20	0.700 (0.6590)	6.9	0.509 (0.6347)	9.5	0.526 (0.6469)	9.2	1.677 (0.6635)	5.8
	35	0.802 (0.9632)	6.1	0.572 (0.8980)	8.5	0.658 (0.9331)	7.4	1.831 (0.9446)	5.3
	50	0.843 (0.9025)	5.8	0.586 (0.9117)	8.3	0.663 (0.9742)	7.3	2.311 (0.9073)	4.2
Fructose/ Glucose	20	0.670 (0.6691)	7.2	0.468 (0.6472)	10.4	0.498 (0.6291)	9.7	1.394 (0.6523)	7.0
	35	0.678 (0.9633)	7.2	0.484 (0.9189)	10.0	0.577 (0.9147)	8.4	1.758 (0.9469)	5.5
	50	0.782 (0.8999)	6.2	0.484 (0.8516)	10.0	0.614 (0.9438)	7.9	2.126 (0.9312)	4.6

^a^ cyanidin-3-*O*-glucoside; ^b^ cyanidin-3-*O*-rutinoside; ^c^ cyanidin-3-*O*-galactoside; ^d^ delphinidin-3-*O*-rutinoside; ^e^ values in parentheses are given for the coefficients of determination; ^f^ reaction rate constant; ^g^ half-life during degradation kinetics of anthocyanins.

**Table 3:** Effect of temperature and selected carbohydrate on activation energy (Ea) values during the degradation reaction of anthocyanins and red dye (a*).

Solvent	Ea (kJ/mol)							
	Cya3Glc ^a^	a*	Cya3Rut ^b^	a*	Cya3Gal ^c^	a*	Del3Rut ^d^	a*
Water	1.49 ± 0.25 (0.9495) ^e^	17.90 ± 0.94 (0.7883)	7.09 ± 0.71 (0.9688)	19.38 ± 2.91 (0.8294)	2.74 ± 0.41 (0.9981)	2.10 ± 0.32 (0.9566)	27.27 ± 4.09 (0.9862)	14.56 ± 2.18 (0.8026)
Glucose	5.11 ± 0.77 (0.8325)	32.91 ± 4.94 (0.8396)	6.12 ± 0.92 (0.8947)	46.69 ± 7.00 (0.9802)	5.97 ± 0.91 (0.9584)	19.77 ± 2.97 (0.8139)	7.14 ± 1.07 (0.8534)	63.57 ± 9.54 (0.7686)
Sucrose	2.54 ± 0.38 (0.9946)	34.81 ± 5.22 (0.9593)	9.93 ± 1.49 (0.8450)	66.30 ± 9.95 (0.9802)	4.82 ± 0.72 (0.8328)	36.57 ± 5.49 (0.8475)	4.00 ± 0.41 (0.8982)	17.76 ± 2.66 (0.8290)
Fructose	4.91 ± 0.74 (0.9468)	50.71 ± 7.61 (0.8668)	3.73 ± 0.56 (0.8938)	68.02 ± 10.20 (0.8737)	6.14 ± 0.92 (0.7965)	31.58 ± 4.74 (0.9495)	8.35 ± 1.25 (0.9218)	32.53 ± 4.88 (0.8073)
Glc/Fru	3.98 ± 0.59 (0.7844)	44.60 ± 6.69 (0.9792)	0.87 ± 0.13 (0.7775)	51.17 ± 7.68 (0.9493)	5.52 ± 0.83 (0.9600)	52.10 ± 7.82 (0.9829)	11.10 ± 1.67 (0.9992)	17.89 ± 2.68 (0.9142)

^a^ cyanidin-3-*O*-glucoside; ^b^ cyanidin-3-*O*-rutinoside; ^c^ cyanidin-3-*O*-galactoside; ^d^ delphinidin-3-*O*-rutinoside; ^e^ values in parentheses are given for the coefficients of determination.

## Data Availability

The original contributions presented in the study are included in the article/Supplementary Material, further inquiries can be directed to the corresponding author.

## Supplementary Materials

The following supporting information can be downloaded at: https://www.mdpi.com/article/10.3390/foods13223628/s1, Table S1: Perception of colour change according to (Shevell, 2004). Adjusted based on the data resulting colorimetric parameters performed in the study.; Figure S1: Chromatograms of anthocyanin standards determined by HPLC/DAD at 100 mg L^−1^. In Figure S1a shows the Cya3Glc standard at a concentration of 100 mg L^−1^ and Figure S1b is the chromatogram of the Cya3Rut standard of the same concentration.; Figure S2: Chromatograms of anthocyanin standards determined by HPLC/DAD at 100 mg L^−1^. In Figure S2a shows the Cya3Gal standard at a concentration of 100 mg L^−1^ and Figure S2b is the chromatogram of the Del3Rut standard of the same concentration.; Figure S3: Arrhenius plots (dependence of rate constants on temperature) for the degradation of anthocyanin Cya3Glc in the presence of selected carbohydrates and correlation with red colour degradation (*a**); Figure S4: Arrhenius plots (dependence of rate constants on temperature) for the degradation of Cya3Rut anthocyanin in the presence of selected carbohydrates and correlation with red colour degradation (*a**); Figure S5: Arrhenius plots (dependence of rate constants on temperature) for the degradation of anthocyanin Cya3Gal in the presence of selected carbohydrates and correlation with red colour degradation (*a**); Figure S6: Arrhenius plots (dependence of rate constants on temperature) for the degradation of Del3Rut anthocyanin in the presence of selected carbohydrates and correlation with red colour degradation (*a**).
