# Supplementary material for: Effect of Various Carbohydrates in Aqueous Solutions on Color Stability and Degradation Kinetics of Selected Anthocyanins During Storage
Source: Foods. 2024 Nov 14;13(22):3628. doi: 10.3390/foods13223628 (PMC11594173; doi:10.3390/foods13223628)
Supplement: Supplementary file 1 [file foods-13-03628-s001.zip › foods-3197673-supplementary.pdf]

Table S.1: Perception of colour change according to (Shevell, 2004). Adjusted based on the data resulting colorimetric parameters performed in the study.

ΔE	Perception of colour change
0 < ΔE < 1	observer does not notice the difference
1 < ΔE < 2	only experienced observer can notice the difference
2 < ΔE < 3.5	inexperienced observer also notices the difference
3.5 < ΔE < 5	the clear color difference is noticed
5 < ΔE	observer notices two different colors

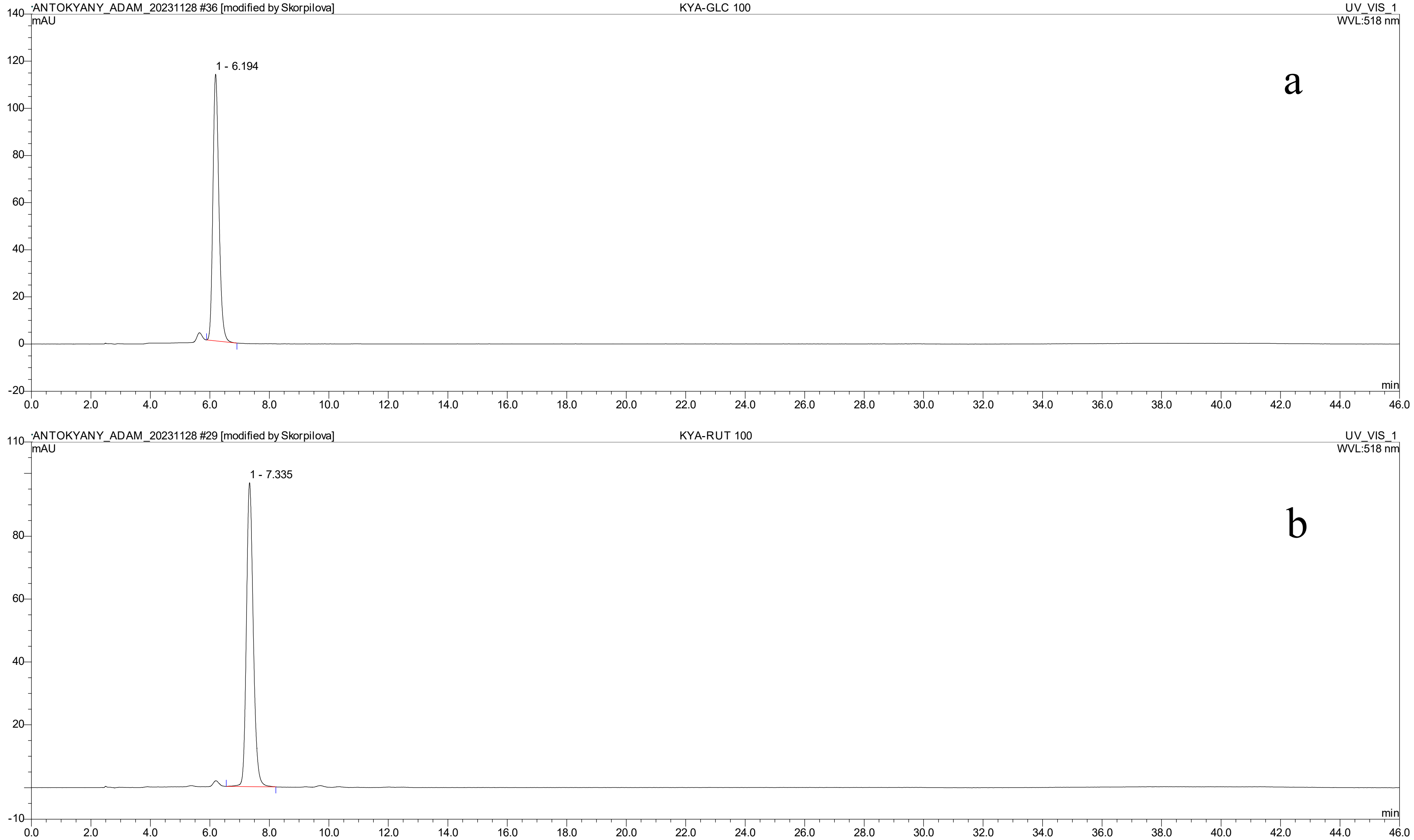

Figure S.1: Chromatograms of anthocyanin standards determined by HPLC/DAD at 100 mg L^{-1}. In Fig. S.1a shows the Cya3Glc standard at a concentration of 100 mg L^{-1} and Fig. S.1b is the chromatogram of the Cya3Rut standard of the same concentration.

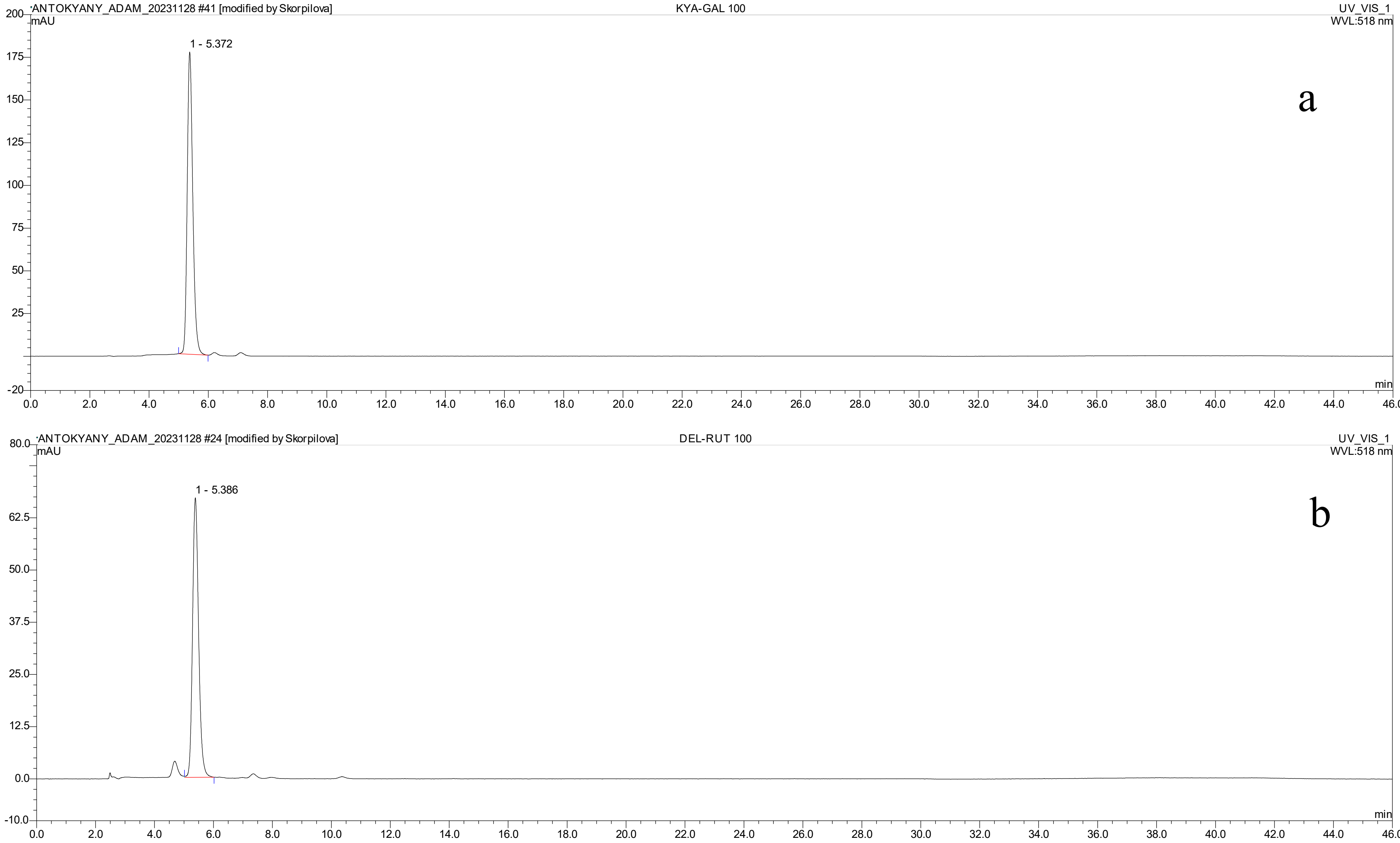

Figure S.2: Chromatograms of anthocyanin standards determined by HPLC/DAD at 100 mg L^{-1}. In Fig. S.2a shows the Cya3Gal standard at a concentration of 100 mg L^{-1} and Fig. S.2b is the chromatogram of the Del3Rut standard of the same concentration.

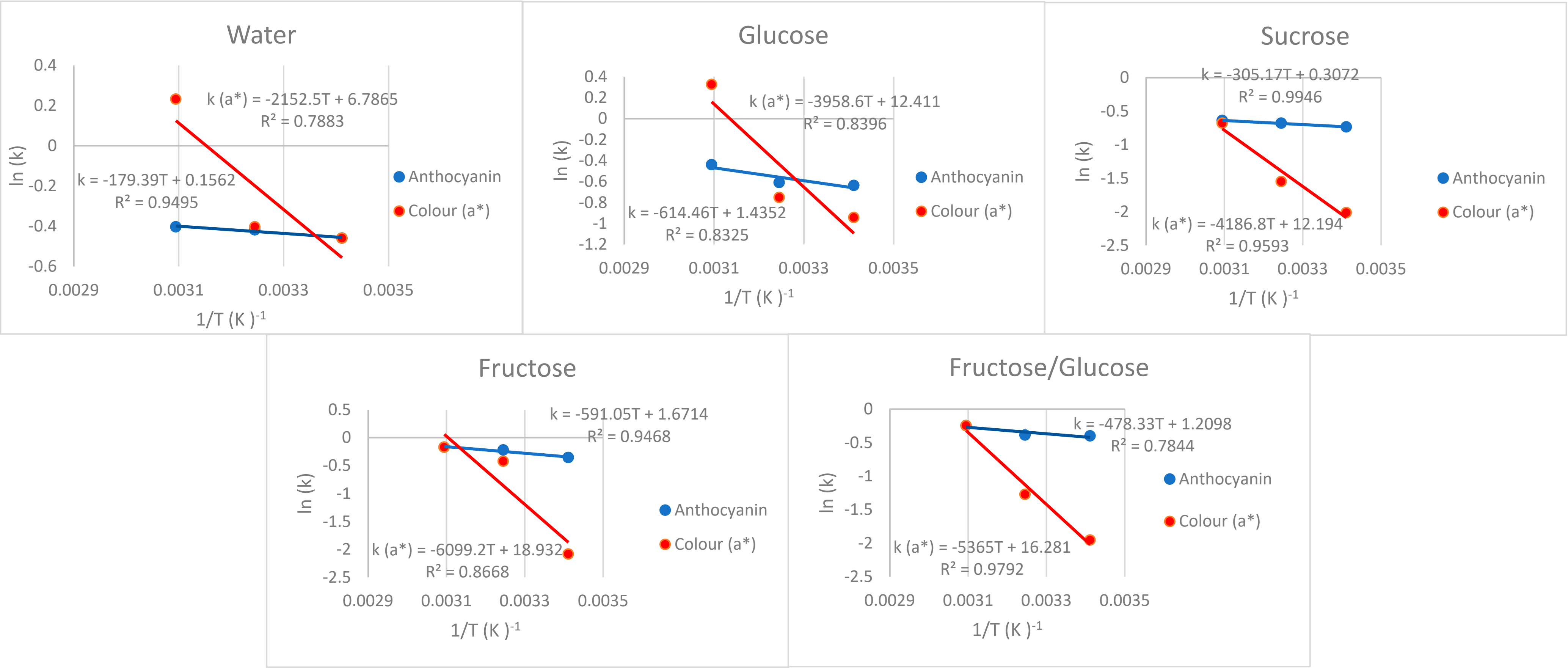

Figure S.3: Arrhenius plots (dependence of rate constants on temperature) for the degradation of anthocyanin Cya3Glc in the presence of selected carbohydrates and correlation with red colour degradation (*a**)

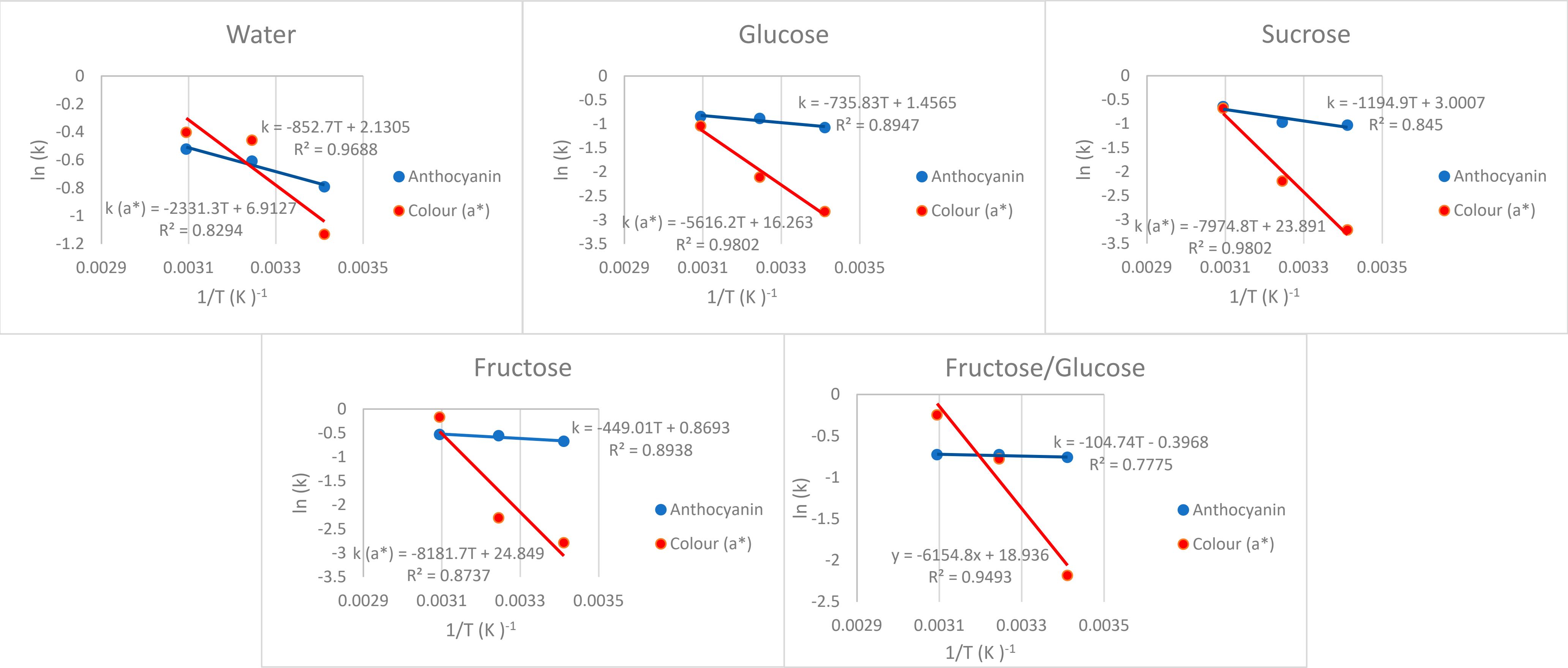

Figure S.4: Arrhenius plots (dependence of rate constants on temperature) for the degradation of Cya3Rut anthocyanin in the presence of selected carbohydrates and correlation with red colour degradation (*a**)

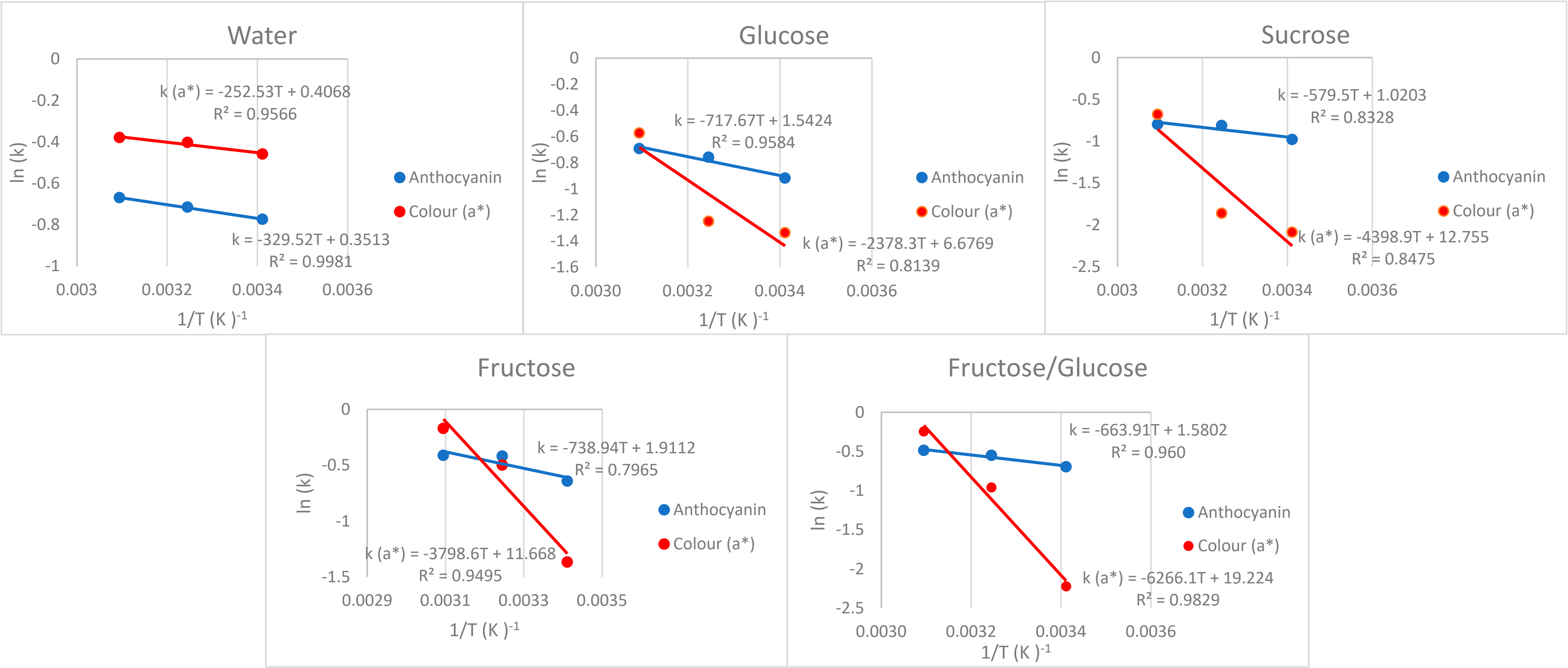

Figure S.5: Arrhenius plots (dependence of rate constants on temperature) for the degradation of anthocyanin Cya3Gal in the presence of selected carbohydrates and correlation with red colour degradation (*a**)

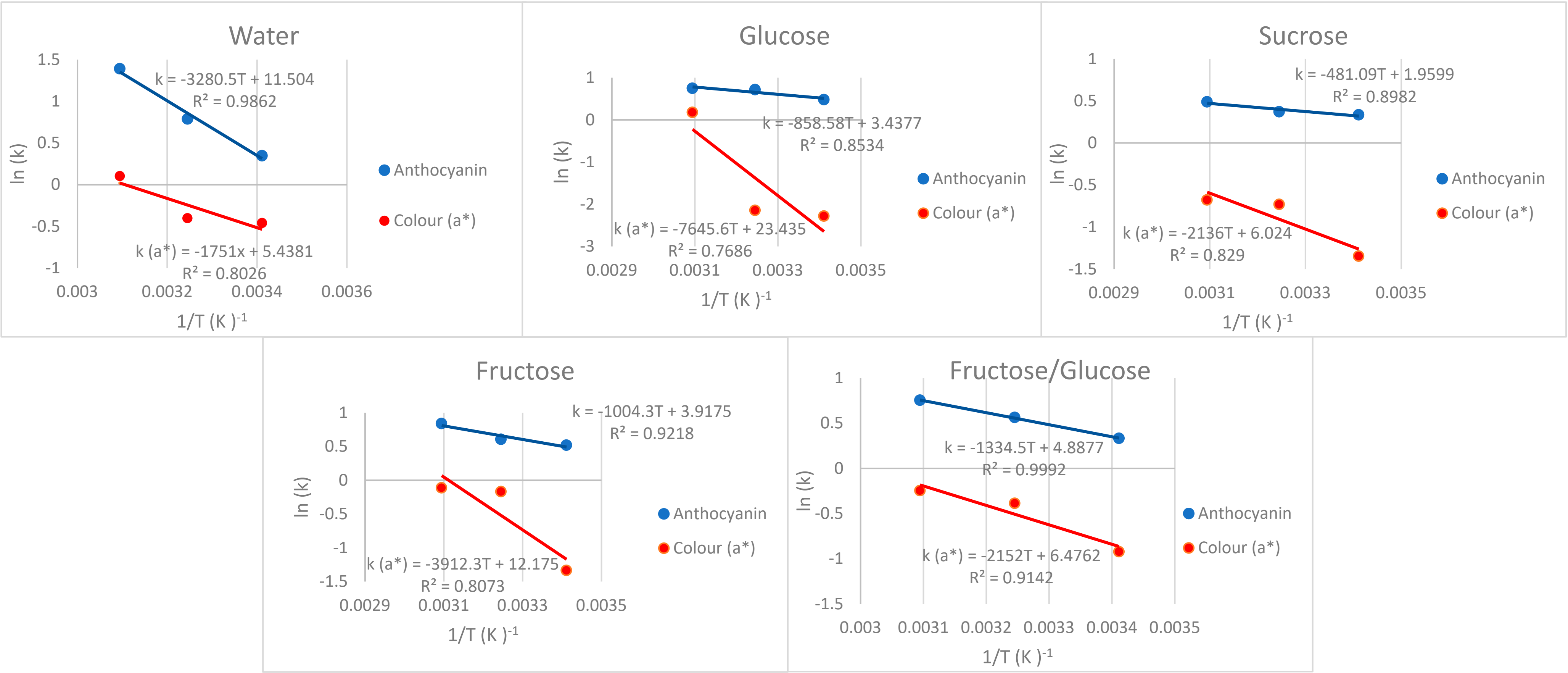

Figure S.6: Arrhenius plots (dependence of rate constants on temperature) for the degradation of Del3Rut anthocyanin in the presence of selected carbohydrates and correlation with red colour degradation (*a**)
